# Faults in Institution Menus

**Published:** 1911-11-25

**Authors:** 


### INSTITUTIONAL HOUSEKEEPING.

(Contributions invited: Workers' Questions will be welcome.)

### Faults in Institution Menus.

A good meal will consist of proteids, fats, carbohydrates, natural salts, and water in such proportions that all the necessary elements are presented without throwing an unfair strain upon any one part of the digestive system, as would be the case if too much meat, or too much sugar, or too much starch were consumed at a time. It must be presented in an attractive form in order that imagination, which plays so important a part in digestion, may lend its aid. And it must present the consumer with quantities sufficient to satisfy the appetite without burdening the digestion. It must never be forgotten that though insufficient supplies of food in course of time render the body ill-nourished and incapable of sustained exertion, the consumption of more food than is needed for the daily renewals and repairs is at once placed to the discredit of the consumer, and is a source of immediate discomfort, leading on possibly towards disease.

Some provision there should always be in hospitals for those members of the staff who come to table tense after some spell of arduous duty, as often happens. When this is the case and no interval of relaxation has intervened before the meal, it is simply to court dyspepsia to bolt a plate full of not too tender meat, followed by some heavy pudding, with perhaps a glass of ale or milk, and then rush back to work. It would be far better under such circumstances to take merely a glass of hot milk with bread and butter and stewed fruit, and postpone the heavy meal even for a day, if need be. The addition of a savoury dish of a light and nourishing nature to the ordinary bill of fare would be a great protection to those who sit down to table "too tired to eat."

